# Courier Review Call-Book

**Published:** 1884-12

**Authors:** 


COURIER REVIEW CALL BOOK, for 1885, being a physican's pocket reference book and visiting list. The following is the table of contents: Almanac, Table of Signs, Preface, Number of Drops in a Fluid Dram, The Metric System, Diagnostic Table of Eruptive Fevers, Poison Treatment, Artificial Respiration, Disinfectants, Posological Table, Doses for Hypodermic Injection, Doses for Inhalation, Comparison of Thermometric Scales, Doses of Medicine for Children, Clinical

Examination of Urine, Diet Table for Diabetics, Prediction of Date of Confinement, Blank Leaves for Visiting List. J. H. Chambers & Co., St. Louis.

